# Supplementary material for: Evaluating Seqstant LiveGene Analysis in real-time assessment of metagenomic next-generation sequencing (mNGS) data from respiratory samples
Source: Infection. 2025 Nov 11;54(2):707–15. doi: 10.1007/s15010-025-02665-y (PMC13021706; doi:10.1007/s15010-025-02665-y)
Supplement: Supplementary file 1 [file 15010_2025_2665_MOESM1_ESM.docx]

**SUPPLEMENT**

**Supplementary data**

**Exclusion criteria from the study**

Out of the initial 384 samples, 49 were excluded, leaving 335 samples for evaluation. The reasons for exclusion were the following:

1. **Sequencing run failure (16 samples)**

One sequencing run involving 16 samples yielded only 4.3 million clusters, significantly below the expected 25 million clusters. This discrepancy indicates a probable error in the loading of the sequencing device, resulting in insufficient data generation for all samples in that run

1. **Underclustering of individual samples (8 samples)**

Eight samples exhibited less than 100,000 clusters, suggesting potential pooling errors during library preparation. These samples were excluded due to the high likelihood of compromised data quality.

1. **Failed sample preparation (20 samples)**

Parts of the first two processed batches failed to produce libraries of sufficient quality for sequencing. Due to limited leftover material, reprocessing was not feasible. Subsequently, library preparation parameters were optimized, successfully resolving the issue in later batches.

1. **Evaluation Deviations and Sample Integrity Concerns (5 samples)**

Five samples were excluded due to deviations in the evaluation process or concerns regarding sample integrity:

- Two samples were not evaluated by one or more medical experts.
- One sample was excluded due to an ambiguous species-level identification of *Fusobacterium sp.*; under the original evaluation scheme, results for this samples would have been in favor of rt-mNGS method.
- One sample showed identical rt-mNGS results to its neighboring sample during processing, while cultivation revealed a distinct microbial profile, indicating a probable sample interchange.
- One wound swab was mistakenly included in the study despite not meeting inclusion criteria.

**LiveGene Analysis Scoring and Performance Evaluation Strategy**

For the evaluation of results, a reported Level of Evidence (LoE) was developed. The LoE is a scoring method that incorporates the most critical characteristics from read alignment:

1. **Unique Hits**: The total number of reads that map uniquely to a specific organism.

2. **Best Hits**: The total number of reads that map to a specific organism with the highest score.

3. **Alignment Quality**: Weighting factors for individual reads, which include the read length after preprocessing and the alignment score.

4. **Genome Coverage**: The uniform distribution of unique hits and best hits across different genomic locations.

5. **Shared Alignments**: The number of reads that are shared between organisms.

The lowest reported LoE is 1 while a LoE of 4 represents the highest evidence for a true hit. A detailed explanation of the different levels of evidence is provided in the supplementary material. Only hits with a reported Level of Evidence (LoE) of 2 or higher (“most likely a true hit”) were considered. The chosen threshold for positivity (LoE ≥ 2) demands a sufficiently high number of reads assigned to a specific organism and a uniform distribution of alignments across the reference genome. The results of rt-mNGS were combined with cultivation results into a single, blinded table that included all considered hits from both methods. This blinded table was evaluated by a medical expert panel consisting of four independent clinical microbiologists and infection specialists. To allow a majority decision, each sample was randomly assigned to three of the four medical experts that assessed the plausibility and clinical relevance of each hit. For each hit deemed clinically relevant and plausible, each method that detected the hit was awarded 1 point. If a hit was deemed clinically relevant but not plausible, each method that detected the hit was deducted 1 point. In all other cases, the method received 0 points. For each sample, a method was awarded a non-inferiority score of 1 if it achieved at least the same score as the other method. The sum of the non-inferiority scores of all samples was used for statistical evaluation. This approach ensures a comprehensive and fair comparison of the rt-mNGS method with traditional cultivation techniques.

**Explanation of the Levels of Evidence**

*LoE 4 – very reliable identification*: The analytical result is very reliable and based on a quantitatively and qualitatively robust data basis. A high number of reads unambiguously assigned to the pathogen is available. The reference genome is covered to a sufficiently high degree so that random hits or artifacts can be excluded. There are also few to no ambiguous reads, which in most cases is due to high sequence similarity to other pathogens.

*LoE 3 – reliable identification*: The analytical result is reliable but based on a quantitatively and/or qualitatively lower data basis than for LoE 4. In general, this results from a lower number of reads and/or an unfavorable distribution of the hits found over the corresponding reference genome.

*LoE 2 – likely true identification*: The analytical result is most likely correct. False positives may occur in a few cases. Reasons for a lower score compared to LoE 3 and LoE 4 include a significantly lower number of reads, clustering in the coverage of the reference genome, or high genetic similarity to another pathogen. In intermediate results, a later report should be awaited if possible. If an immediate treatment decision needs to be made, the pathogen should additionally be detected via complementary technology and/or it should be verified that the pathogen found fits the context of the patient's clinical course (symptoms, history of disease, earlier diagnoses, etc.).

*LoE 1 – unreliable identification*: There are indications in the genetic data that point to the reported pathogen, for example individual reads that could be clearly assigned to the pathogen. However, there are more false-positive results, for example due to contamination or similarities to pathogens not included in the database. In the case of intermediate results, a later report should be awaited. If an immediate treatment decision must be made, the result should be considered only as an investigative guide. The treatment decision should be made in the knowledge that in many cases it may be a false-positive hit.

*LoE 0 – no identification*: Identifications with LoE 0 are not reported.

The precise methodology for calculating the Level of Evidence in the Seqstant LiveGene Analysis software is proprietary information owned by Seqstant GmbH.

**Statistical calculation for non-inferiority**

The statistical evaluation of the study was performed as a binary non-inferiority study. The study aimed at a proof on non-inferiority with a non-inferiority limit of 10% ($\delta=10$). The proof of non-inferiority is performed with a significance level $\alpha=0.05$ and a statistical power $1-\beta=0.8$. A total number of $n=335$ clinical samples from the human respiratory tract have been evaluated. Results for rt-mNGS have been generated with the commercial software Seqstant LiveGene Analysis v1.0.0.

For comparison purposes, non-inferior performance was defined as the detection of the same or more clinically relevant microbial species compared to each other. The success rate of a method is the percentage of non-inferior samples when compared to the other method. The success rate of gold-standard cultivation ($\pi_{s}$) and rt-mNGS ($\pi_{e}$) is thereby determined by:

$\pi_{s} = \frac{p_{s}}{n}\cdot100$

$\pi_{e}= \frac{p_{e}}{n}\cdot100$

, where $p_{s}$ is the number of non-inferior samples of gold-standard cultivation and $p_{e}$ is the number of non-inferior samples of rt-mNGS.

Evaluation of results showed a non-inferiority of rt-mNGS for 294 samples, whereas cultivation showed non-inferiority for 281 samples. According to these evaluation results, rt-mNGS shows a success rate of $\pi_{e} = \frac{294}{335}\cdot100 \sim87.7612$ and cultivation shows a success rate of $\pi_{s} = \frac{281}{335}\cdot100 \sim83.8806$.

Under a normal distribution approximation, the required sample count for the proof of significance of non-inferiority is given by the following formula using the parameters specified above [1,2]:

$$n\geq\frac{f\left( \alpha, \beta\right)\cdot(\pi_{s}\cdot\left( 100-\pi_{s} \right)+\pi_{e}\cdot\left( 100-\pi_{e} \right))}{{(\pi_{s}-\pi_{e}-\delta)}^{2}}$$

, where

$$f\left( \alpha, \beta\right)={(\Phi^{-1}\left( \alpha\right)+\Phi^{-1}\left( \beta\right))}^{2}$$

$$= \left( \Phi^{-1}\left( 0.05 \right)+ \Phi^{-1}\left( 0.2 \right) \right)^{2}$$

$$\sim\left( -1.6449 - 0.8416 \right)^{2}$$

$$\sim6.1827$$

, and $\Phi^{-1}$ is the cumulative distribution function of the standard normal distribution. The values can, for example, be determined from a table of standard normal distribution. Intermediate values can be approximated by linear interpolation. The values used in the calculations have been determined using the qnorm() function of the statistics software R with parameters mean=0 and sd=1 that correspond to the standard normal distribution [3].

With the evaluated numbers, it holds:

$$n=335 \geq\frac{f\left( \alpha, \beta\right)\cdot(\pi_{s}\cdot\left( 100-\pi_{s} \right)+\pi_{e}\cdot\left( 100-\pi_{e} \right))}{{(\pi_{s}-\pi_{e}-d)}^{2}}$$

$$\sim\frac{6.1827\cdot(83.8806\cdot\left( 100-83.8806 \right)+87.7612\cdot\left( 100-87.7612 \right))}{{(83.8806-87.7612-10)}^{2}}$$

$$\sim77.8552$$

$$∎$$

Thus, non-inferiority of the rt-mNGS approach over gold-standard cultivation was shown with a non-inferiority limit of 10%, a significance level of 0.05 and a power of 0.8.

References:

[1] Blackwelder WC. “Proving the Null Hypothesis” in Clinical Trials. Control. Clin. Trials; 3:345-353

[2] Sealed Envelope Ltd. 2012. Power calculator for binary outcome non-inferiority trial. [Online] Available from: https://www.sealedenvelope.com/power/binary-noninferior/ [Accessed Feb 23, 2022]

[3] R Core Team (2018). R: A language and environment for statistical computing. R Foundation for Statistical Computing, Vienna, Austria. URL: https://www.R-project.org/. [R version 3.4.4 (2018-03-15)]

Supplementary Table 1. Cohort used in the study.

| StudienNr | sample type | Gender | Age | Excluded | Reason of exclusion | Score Seqstant | Score Culture |
| --- | --- | --- | --- | --- | --- | --- | --- |
| CE_001 | Tracheal secretion | male | 63 | 0 |  | 1 | 1 |
| CE_002 | Tracheal secretion | male | 72 | 1 | Failed sample preparation |  |  |
| CE_003 | Tracheal secretion | male | 72 | 1 | Failed sample preparation |  |  |
| CE_004 | Tracheal secretion | male | 75 | 1 | Failed sample preparation |  |  |
| CE_005 | Tracheal secretion | male | 73 | 1 | Failed sample preparation |  |  |
| CE_006 | Tracheal secretion | male | 78 | 1 | Failed sample preparation |  |  |
| CE_007 | Tracheal secretion | male | 83 | 1 | Failed sample preparation |  |  |
| CE_008 | Tracheal secretion | male | 0 | 1 | Failed sample preparation |  |  |
| CE_009 | Tracheal secretion | female | 0 | 1 | Failed sample preparation |  |  |
| CE_010 | Tracheal secretion | male | 88 | 0 |  | 1 | 1 |
| CE_011 | Tracheal secretion | male | 56 | 1 | Failed sample preparation |  |  |
| CE_012 | Tracheal secretion | male | 67 | 1 | Failed sample preparation |  |  |
| CE_013 | Tracheal secretion | male | 70 | 1 | Failed sample preparation |  |  |
| CE_014 | Tracheal secretion | male | 68 | 0 |  | 1 | 1 |
| CE_015 | Sputum | male | 70 | 1 | Failed sample preparation |  |  |
| CE_016 | Tracheal secretion | male | 66 | 0 |  | 1 | 0 |
| CE_017 | Bronchial lavage | male | 56 | 0 |  | 1 | 1 |
| CE_018 | Bronchial lavage | male | 56 | 1 | Failed sample preparation |  |  |
| CE_019 | Tracheal secretion | female | 69 | 0 |  | 0 | 1 |
| CE_020 | Tracheal secretion | male | 48 | 0 |  | 1 | 1 |
| CE_021 | Tracheal secretion | male | 50 | 1 | Failed sample preparation |  |  |
| CE_022 | Tracheal secretion | female | 63 | 0 |  | 0 | 1 |
| CE_023 | Tracheal secretion | male | 58 | 1 | Failed sample preparation |  |  |
| CE_024 | Bronchial lavage | male | 75 | 1 | Failed sample preparation |  |  |
| CE_025 | Bronchial lavage | female | 66 | 1 | Failed sample preparation |  |  |
| CE_026 | Bronchial lavage | male | 75 | 0 |  | 1 | 0 |
| CE_027 | Tracheal secretion | male | 58 | 0 |  | 1 | 1 |
| CE_028 | Bronchial lavage | female | 59 | 1 | Failed sample preparation |  |  |
| CE_029 | Bronchial secretion | female | 87 | 1 | Failed sample preparation |  |  |
| CE_030 | Bronchial secretion | female | 87 | 0 |  | 1 | 0 |
| CE_031 | Tracheal secretion | female | 88 | 1 | Sample Integrity (similar results to CE_030) |  |  |
| CE_032 | Bronchial lavage | male | 38 | 1 | Failed sample preparation |  |  |
| CE_033 | Tracheal secretion | male | 75 | 0 |  | 1 | 0 |
| CE_034 | Tracheal secretion | male | 87 | 0 |  | 1 | 1 |
| CE_035 | Sputum | male | 79 | 0 |  | 0 | 1 |
| CE_036 | Tracheal secretion | female | 0 | 0 |  | 1 | 1 |
| CE_037 | Tracheal secretion | female | 6 | 0 |  | 1 | 1 |
| CE_038 | Bronchial lavage | male | 64 | 0 |  | 1 | 0 |
| CE_039 | Bronchial lavage | male | 56 | 0 |  | 1 | 1 |
| CE_040 | Bronchial secretion | male | 56 | 0 |  | 1 | 0 |
| CE_041 | Bronchial lavage | female | 79 | 0 |  | 1 | 1 |
| CE_042 | Tracheal secretion | male | 62 | 0 |  | 0 | 1 |
| CE_043 | Tracheal secretion | male | 84 | 0 |  | 1 | 0 |
| CE_044 | Tracheal secretion | male | 70 | 0 |  | 1 | 1 |
| CE_045 | Tracheal secretion | female | 0 | 0 |  | 1 | 0 |
| CE_046 | Bronchial lavage | male | 58 | 0 |  | 1 | 1 |
| CE_047 | Bronchial lavage | male | 58 | 0 |  | 1 | 1 |
| CE_048 | Tracheal secretion | male | 77 | 0 |  | 1 | 1 |
| CE_049 | Bronchial lavage | male | 75 | 0 |  | 1 | 1 |
| CE_050 | Bronchial lavage | female | 69 | 0 |  | 1 | 0 |
| CE_051 | Bronchial lavage | male | 69 | 0 |  | 1 | 1 |
| CE_052 | Bronchial lavage | female | 69 | 0 |  | 1 | 0 |
| CE_053 | Tracheal secretion | female | 52 | 0 |  | 1 | 1 |
| CE_054 | Tracheal secretion | female | 66 | 0 |  | 1 | 1 |
| CE_055 | Bronchial secretion | female | 73 | 0 |  | 1 | 1 |
| CE_056 | Bronchial lavage | male | 59 | 0 |  | 1 | 0 |
| CE_057 | Bronchial lavage | male | 56 | 0 |  | 1 | 1 |
| CE_058 | Bronchial secretion | male | 62 | 0 |  | 1 | 1 |
| CE_059 | Bronchial secretion | male | 60 | 0 |  | 1 | 1 |
| CE_060 | Bronchial secretion | male | 51 | 0 |  | 1 | 1 |
| CE_061 | Bronchial secretion | male | 52 | 0 |  | 1 | 1 |
| CE_062 | Bronchial secretion | female | 44 | 0 |  | 1 | 1 |
| CE_063 | Bronchial secretion | female | 70 | 0 |  | 1 | 1 |
| CE_064 | Bronchial secretion | male | 43 | 0 |  | 1 | 0 |
| CE_065 | Bronchial secretion | female | 72 | 0 |  | 0 | 1 |
| CE_066 | Bronchial secretion | male | 60 | 0 |  | 1 | 1 |
| CE_067 | Bronchial lavage | male | 73 | 0 |  | 1 | 1 |
| CE_068 | Bronchial lavage | female | 52 | 0 |  | 1 | 1 |
| CE_069 | Bronchial secretion | female | 61 | 0 |  | 1 | 1 |
| CE_070 | swab |  |  | 1 | Wrong labelling |  |  |
| CE_071 | Tracheal secretion | male | 56 | 0 |  | 1 | 1 |
| CE_072 | Tracheal secretion | female | 71 | 0 |  | 1 | 1 |
| CE_073 | Bronchial lavage | male | 58 | 0 |  | 1 | 1 |
| CE_074 | Tracheal secretion | male | 64 | 0 |  | 1 | 0 |
| CE_075 | Bronchial lavage | male | 58 | 0 |  | 1 | 1 |
| CE_076 | Bronchial secretion | male | 50 | 0 |  | 1 | 1 |
| CE_077 | Tracheal secretion | male | 10 | 0 |  | 1 | 0 |
| CE_078 | Bronchial lavage | male | 61 | 0 |  | 1 | 1 |
| CE_079 | Bronchial secretion | female | 55 | 0 |  | 1 | 0 |
| CE_080 | Tracheal secretion | male | 68 | 0 |  | 1 | 1 |
| CE_081 | Tracheal secretion | male | 78 | 0 |  | 1 | 1 |
| CE_082 | Tracheal secretion | male | 78 | 0 |  | 1 | 1 |
| CE_083 | Bronchial lavage | male | 70 | 0 |  | 0 | 1 |
| CE_084 | Bronchial lavage | female | 59 | 0 |  | 1 | 1 |
| CE_085 | Bronchial lavage | male | 70 | 0 |  | 1 | 0 |
| CE_086 | Bronchial lavage | female | 48 | 0 |  | 1 | 1 |
| CE_087 | Bronchial lavage | female | 48 | 0 |  | 1 | 1 |
| CE_088 | Bronchial lavage | female | 69 | 0 |  | 1 | 0 |
| CE_089 | Bronchial lavage | female | 59 | 0 |  | 1 | 1 |
| CE_090 | Tracheal secretion | female | 0 | 0 |  | 1 | 0 |
| CE_091 | Tracheal secretion | female | 72 | 0 |  | 1 | 0 |
| CE_092 | Tracheal secretion | male | 38 | 1 | Underclustering (<100,000 clusters) |  |  |
| CE_093 | Bronchial lavage | male | 77 | 0 |  | 0 | 1 |
| CE_094 | Tracheal secretion | male | 48 | 0 |  | 1 | 0 |
| CE_095 | Bronchial secretion | male | 51 | 0 |  | 1 | 1 |
| CE_096 | Bronchial secretion | female | 47 | 0 |  | 1 | 0 |
| CE_097 | Tracheal secretion | female | 6 | 0 |  | 1 | 1 |
| CE_098 | Tracheal secretion | female | 0 | 0 |  | 1 | 1 |
| CE_099 | Tracheal secretion | male | 72 | 0 |  | 1 | 1 |
| CE_100 | Tracheal secretion | male | 87 | 0 |  | 1 | 0 |
| CE_101 | Tracheal secretion | male | 75 | 0 |  | 1 | 1 |
| CE_102 | Tracheal secretion | male | 50 | 0 |  | 1 | 1 |
| CE_103 | Tracheal secretion | male | 56 | 0 |  | 1 | 1 |
| CE_104 | Sputum | male | 80 | 0 |  | 1 | 0 |
| CE_105 | Tracheal secretion | male | 73 | 0 |  | 1 | 1 |
| CE_106 | Tracheal secretion | male | 83 | 0 |  | 1 | 0 |
| CE_107 | Tracheal secretion | male | 68 | 0 |  | 1 | 1 |
| CE_108 | Bronchial secretion | male | 60 | 0 |  | 1 | 1 |
| CE_109 | Tracheal secretion | male | 78 | 0 |  | 1 | 1 |
| CE_110 | Tracheal secretion | male | 78 | 0 |  | 1 | 1 |
| CE_111 | Bronchial secretion | male | 34 | 0 |  | 1 | 1 |
| CE_112 | Tracheal secretion | male | 48 | 0 |  | 1 | 0 |
| CE_113 | Tracheal secretion | male | 63 | 0 |  | 1 | 1 |
| CE_114 | Tracheal secretion | male | 68 | 0 |  | 0 | 1 |
| CE_115 | Tracheal secretion | male | 91 | 0 |  | 1 | 1 |
| CE_116 | Bronchial secretion | female | 47 | 0 |  | 0 | 1 |
| CE_117 | Bronchial secretion | male | 70 | 0 |  | 1 | 1 |
| CE_118 | Bronchial secretion | male | 84 | 0 |  | 0 | 1 |
| CE_119 | Bronchial secretion | female | 64 | 0 |  | 1 | 1 |
| CE_120 | Bronchial secretion | female | 52 | 0 |  | 1 | 1 |
| CE_121 | Tracheal secretion | male | 75 | 0 |  | 1 | 0 |
| CE_122 | Tracheal secretion | male | 88 | 0 |  | 1 | 1 |
| CE_123 | Tracheal secretion | male | 68 | 0 |  | 0 | 1 |
| CE_124 | Bronchial lavage | female | 88 | 0 |  | 1 | 1 |
| CE_125 | Bronchial secretion | male | 72 | 0 |  | 0 | 1 |
| CE_126 | Bronchial secretion | female | 70 | 0 |  | 1 | 1 |
| CE_127 | Tracheal secretion | male | 77 | 0 |  | 0 | 1 |
| CE_128 | Tracheal secretion | male | 69 | 0 |  | 1 | 1 |
| CE_129 | Tracheal secretion | female | 0 | 0 |  | 1 | 0 |
| CE_130 | Bronchial lavage | male | 67 | 0 |  | 1 | 1 |
| CE_131 | Tracheal secretion | female | 69 | 0 |  | 1 | 1 |
| CE_132 | Bronchial lavage | female | 55 | 0 |  | 1 | 1 |
| CE_133 | Tracheal secretion | male | 60 | 0 |  | 1 | 1 |
| CE_134 | Bronchial lavage | female | 65 | 0 |  | 1 | 0 |
| CE_135 | Bronchial secretion | female | 73 | 0 |  | 1 | 1 |
| CE_136 | Bronchial secretion | male | 75 | 0 |  | 1 | 1 |
| CE_137 | Bronchial secretion | male | 26 | 0 |  | 1 | 1 |
| CE_138 | Bronchial secretion | male | 64 | 0 |  | 1 | 1 |
| CE_139 | Bronchial secretion | female | 66 | 0 |  | 1 | 0 |
| CE_140 | Bronchial lavage | male | 85 | 0 |  | 1 | 0 |
| CE_141 | Bronchial lavage | female | 73 | 0 |  | 1 | 0 |
| CE_142 | Bronchial lavage | female | 37 | 0 |  | 1 | 0 |
| CE_143 | Bronchial lavage | female | 37 | 0 |  | 1 | 1 |
| CE_144 | Bronchial secretion | male | 57 | 0 |  | 1 | 1 |
| CE_145 | Tracheal secretion | female | 75 | 1 | Sequencing run failure |  |  |
| CE_146 | Tracheal secretion | female | 81 | 1 | Sequencing run failure |  |  |
| CE_147 | Tracheal secretion | female | 72 | 1 | Sequencing run failure |  |  |
| CE_148 | Tracheal secretion | male | 68 | 1 | Sequencing run failure |  |  |
| CE_149 | Sputum | male | 63 | 1 | Sequencing run failure |  |  |
| CE_150 | Bronchial secretion | male | 63 | 1 | Sequencing run failure |  |  |
| CE_151 | Bronchial secretion | male | 47 | 1 | Sequencing run failure |  |  |
| CE_152 | Bronchial secretion | male | 47 | 1 | Sequencing run failure |  |  |
| CE_153 | Bronchial secretion | female | 56 | 1 | Sequencing run failure |  |  |
| CE_154 | Tracheal secretion | male | 50 | 1 | Sequencing run failure |  |  |
| CE_155 | Tracheal secretion | female | 50 | 1 | Sequencing run failure |  |  |
| CE_156 | Tracheal secretion | male | 70 | 1 | Sequencing run failure |  |  |
| CE_157 | Bronchial secretion | male | 68 | 1 | Sequencing run failure |  |  |
| CE_158 | Bronchial secretion | female | 60 | 1 | Sequencing run failure |  |  |
| CE_159 | Bronchial secretion | female | 75 | 1 | Sequencing run failure |  |  |
| CE_160 | Bronchial lavage | female | 80 | 1 | Sequencing run failure |  |  |
| CE_161 | Bronchial lavage | female | 80 | 0 |  | 1 | 1 |
| CE_162 | Bronchial lavage | female | 56 | 0 |  | 1 | 1 |
| CE_163 | Bronchial secretion | male | 82 | 0 |  | 1 | 1 |
| CE_164 | Bronchial secretion | male | 81 | 0 |  | 1 | 1 |
| CE_165 | Bronchial secretion | male | 75 | 0 |  | 1 | 1 |
| CE_166 | Tracheal secretion | male | 51 | 0 |  | 1 | 1 |
| CE_167 | Bronchial lavage | male | 75 | 0 |  | 1 | 0 |
| CE_168 | Bronchial secretion | female | 75 | 0 |  | 0 | 1 |
| CE_169 | Tracheal secretion | female | 69 | 0 |  | 0 | 1 |
| CE_170 | Tracheal secretion | female | 71 | 0 |  | 1 | 1 |
| CE_171 | Tracheal secretion | male | 80 | 0 |  | 1 | 1 |
| CE_172 | Tracheal secretion | male | 83 | 0 |  | 1 | 0 |
| CE_173 | Tracheal secretion | male | 68 | 0 |  | 1 | 1 |
| CE_174 | Bronchial lavage | male | 38 | 0 |  | 1 | 1 |
| CE_175 | Tracheal secretion | male | 72 | 0 |  | 1 | 0 |
| CE_176 | Tracheal secretion | male | 56 | 0 |  | 1 | 0 |
| CE_177 | Tracheal secretion | male | 75 | 0 |  | 1 | 1 |
| CE_178 | Tracheal secretion | male | 75 | 0 |  | 1 | 1 |
| CE_179 | Tracheal secretion | male | 69 | 0 |  | 1 | 0 |
| CE_180 | Tracheal secretion | male | 66 | 0 |  | 0 | 1 |
| CE_181 | Bronchial lavage | male | 75 | 0 |  | 1 | 1 |
| CE_182 | Bronchial lavage | male | 75 | 0 |  | 1 | 1 |
| CE_183 | Bronchial lavage | male | 56 | 0 |  | 1 | 1 |
| CE_184 | Tracheal secretion | female | 83 | 0 |  | 1 | 1 |
| CE_185 | Tracheal secretion | female | 6 | 0 |  | 1 | 1 |
| CE_186 | Tracheal secretion | male | 0 | 0 |  | 1 | 1 |
| CE_187 | Tracheal secretion | male | 63 | 0 |  | 1 | 1 |
| CE_188 | Tracheal secretion | male | 50 | 0 |  | 1 | 1 |
| CE_189 | Tracheal secretion | male | 71 | 0 |  | 0 | 1 |
| CE_190 | Bronchial secretion | female | 69 | 0 |  | 1 | 1 |
| CE_191 | Bronchial secretion | male | 78 | 0 |  | 1 | 1 |
| CE_192 | Tracheal secretion | male | 89 | 0 |  | 0 | 1 |
| CE_193 | Tracheal secretion | male | 82 | 0 |  | 1 | 1 |
| CE_194 | Sputum | male | 78 | 0 |  | 1 | 1 |
| CE_195 | Tracheal secretion | male | 81 | 0 |  | 1 | 1 |
| CE_196 | Tracheal secretion | male | 64 | 0 |  | 0 | 1 |
| CE_197 | Bronchial lavage | male | 77 | 1 | Underclustering (<100,000 clusters) |  |  |
| CE_198 | Bronchial lavage | male | 68 | 0 |  | 0 | 1 |
| CE_199 | Bronchial lavage | male | 68 | 0 |  | 1 | 1 |
| CE_200 | Bronchial lavage | male | 73 | 0 |  | 1 | 1 |
| CE_201 | Bronchial secretion | female | 22 | 0 |  | 0 | 1 |
| CE_202 | Bronchial secretion | female | 72 | 0 |  | 1 | 1 |
| CE_203 | Bronchial secretion | female | 69 | 0 |  | 1 | 1 |
| CE_204 | Bronchial secretion | female | 58 | 0 |  | 1 | 1 |
| CE_205 | Tracheal secretion | female | 72 | 0 |  | 1 | 1 |
| CE_206 | Bronchial secretion | male | 64 | 0 |  | 1 | 1 |
| CE_207 | Bronchial secretion | female | 61 | 0 |  | 1 | 1 |
| CE_208 | Tracheal secretion | male | 0 | 0 |  | 1 | 1 |
| CE_209 | Bronchial lavage | female | 59 | 0 |  | 0 | 1 |
| CE_210 | Bronchial lavage | female | 59 | 0 |  | 0 | 1 |
| CE_211 | Tracheal secretion | male | 1 | 0 |  | 1 | 1 |
| CE_212 | Bronchial lavage | male | 73 | 0 |  | 1 | 1 |
| CE_213 | Tracheal secretion | male | 94 | 1 | Underclustering (<100,000 clusters) |  |  |
| CE_214 | Bronchial secretion | female | 37 | 0 |  | 1 | 1 |
| CE_215 | Tracheal secretion | male | 78 | 0 |  | 1 | 1 |
| CE_216 | Bronchial secretion | female | 64 | 0 |  | 1 | 1 |
| CE_217 | Tracheal secretion | male | 63 | 0 |  | 1 | 1 |
| CE_218 | Bronchial lavage | male | 73 | 0 |  | 1 | 1 |
| CE_219 | Tracheal secretion | male | 56 | 0 |  | 0 | 1 |
| CE_220 | Bronchial secretion | female | 55 | 0 |  | 1 | 0 |
| CE_221 | Bronchial lavage | female | 36 | 0 |  | 1 | 1 |
| CE_222 | Bronchial secretion | female | 43 | 0 |  | 1 | 1 |
| CE_223 | Bronchial secretion | male | 72 | 0 |  | 1 | 1 |
| CE_224 | Bronchial secretion | female | 60 | 0 |  | 1 | 1 |
| CE_225 | Bronchial lavage | male | 47 | 0 |  | 1 | 1 |
| CE_226 | Tracheal secretion | male | 66 | 0 |  | 1 | 1 |
| CE_227 | Bronchial secretion | female | 73 | 0 |  | 0 | 1 |
| CE_228 | Bronchial secretion | female | 50 | 0 |  | 1 | 0 |
| CE_229 | Bronchial secretion | male | 69 | 0 |  | 0 | 1 |
| CE_230 | Tracheal secretion | female | 59 | 0 |  | 1 | 1 |
| CE_231 | Tracheal secretion | female | 79 | 0 |  | 1 | 1 |
| CE_232 | Bronchial secretion | male | 58 | 0 |  | 1 | 1 |
| CE_233 | Bronchial lavage | male | 66 | 0 |  | 1 | 1 |
| CE_234 | Bronchial secretion | male | 62 | 0 |  | 1 | 1 |
| CE_235 | Sputum | male | 0 | 0 |  | 1 | 1 |
| CE_236 | Tracheal secretion | female | 48 | 1 | Underclustering (<100,000 clusters) |  |  |
| CE_237 | Tracheal secretion | male | 66 | 0 |  | 1 | 1 |
| CE_238 | Tracheal secretion | female | 47 | 0 |  | 1 | 1 |
| CE_239 | Tracheal secretion | female | 83 | 0 |  | 1 | 1 |
| CE_240 | Bronchial secretion | female | 57 | 0 |  | 1 | 1 |
| CE_241 | Tracheal secretion | male | 27 | 0 |  | 1 | 1 |
| CE_242 | Bronchial secretion | male | 63 | 0 |  | 1 | 1 |
| CE_243 | Tracheal secretion | female | 60 | 0 |  | 0 | 1 |
| CE_244 | Tracheal secretion | male | 68 | 0 |  | 1 | 1 |
| CE_245 | Tracheal secretion | male | 75 | 0 |  | 1 | 1 |
| CE_246 | Tracheal secretion | male | 68 | 0 |  | 1 | 1 |
| CE_247 | Tracheal secretion | male | 56 | 0 |  | 0 | 1 |
| CE_248 | Tracheal secretion | male | 0 | 0 |  | 1 | 1 |
| CE_249 | Tracheal secretion | male | 41 | 0 |  | 0 | 1 |
| CE_250 | Tracheal secretion | male | 74 | 0 |  | 1 | 1 |
| CE_251 | Tracheal secretion | male | 58 | 0 |  | 1 | 1 |
| CE_252 | Tracheal secretion | female | 73 | 0 |  | 1 | 1 |
| CE_253 | Tracheal secretion | female | 83 | 0 |  | 0 | 1 |
| CE_254 | Tracheal secretion | female | 64 | 0 |  | 1 | 1 |
| CE_255 | Bronchial lavage | female | 68 | 0 |  | 1 | 1 |
| CE_256 | Tracheal secretion | male | 69 | 0 |  | 1 | 0 |
| CE_257 | Bronchial secretion | male | 83 | 0 |  | 0 | 1 |
| CE_258 | Bronchial lavage | female | 88 | 0 |  | 1 | 1 |
| CE_259 | Tracheal secretion | male | 80 | 0 |  | 1 | 1 |
| CE_260 | Bronchial secretion | female | 70 | 0 |  | 1 | 1 |
| CE_261 | Bronchial secretion | female | 82 | 0 |  | 1 | 0 |
| CE_262 | Bronchial secretion | female | 58 | 0 |  | 0 | 1 |
| CE_263 | Bronchial lavage | female | 68 | 0 |  | 1 | 1 |
| CE_264 | Bronchial secretion | male | 73 | 0 |  | 1 | 1 |
| CE_265 | Tracheal secretion | female | 6 | 0 |  | 1 | 1 |
| CE_266 | Tracheal secretion | female | 4 | 0 |  | 1 | 0 |
| CE_267 | Tracheal secretion | male | 81 | 0 |  | 1 | 1 |
| CE_268 | Tracheal secretion | male | 78 | 0 |  | 1 | 1 |
| CE_269 | Tracheal secretion | male | 62 | 0 |  | 1 | 1 |
| CE_270 | Tracheal secretion | female | 63 | 0 |  | 1 | 1 |
| CE_271 | Bronchial secretion | male | 75 | 0 |  | 1 | 1 |
| CE_272 | Tracheal secretion | male | 58 | 0 |  | 1 | 1 |
| CE_273 | Bronchial secretion | male | 58 | 0 |  | 1 | 1 |
| CE_274 | Bronchial secretion | male | 58 | 0 |  | 1 | 1 |
| CE_275 | Tracheal secretion | male | 79 | 0 |  | 1 | 1 |
| CE_276 | Tracheal secretion | female | 72 | 0 |  | 1 | 1 |
| CE_277 | Bronchial secretion | male | 58 | 0 |  | 1 | 1 |
| CE_278 | Bronchial secretion | female | 53 | 0 |  | 1 | 1 |
| CE_279 | Bronchial secretion | female | 55 | 0 |  | 1 | 1 |
| CE_280 | Bronchial secretion | male | 74 | 0 |  | 1 | 1 |
| CE_281 | Bronchial secretion | male | 53 | 0 |  | 1 | 1 |
| CE_282 | Bronchial lavage | female | 4 | 0 |  | 1 | 1 |
| CE_283 | Tracheal secretion | male | 77 | 0 |  | 1 | 1 |
| CE_284 | Tracheal secretion | male | 59 | 0 |  | 1 | 1 |
| CE_285 | Tracheal secretion | female | 47 | 0 |  | 0 | 1 |
| CE_286 | Bronchial lavage | male | 78 | 0 |  | 1 | 1 |
| CE_287 | Bronchial secretion | female | 67 | 0 |  | 1 | 1 |
| CE_288 | Bronchial secretion | male | 35 | 0 |  | 1 | 1 |
| CE_289 | Tracheal secretion | female | 69 | 0 |  | 1 | 1 |
| CE_290 | Tracheal secretion | female | 53 | 0 |  | 1 | 1 |
| CE_291 | Tracheal secretion | female | 54 | 0 |  | 1 | 0 |
| CE_292 | Bronchial secretion | male | 59 | 0 |  | 1 | 1 |
| CE_293 | Tracheal secretion | female | 59 | 0 |  | 1 | 0 |
| CE_294 | Bronchial secretion | female | 68 | 0 |  | 1 | 0 |
| CE_295 | Bronchial secretion | male | 76 | 0 |  | 1 | 0 |
| CE_296 | Bronchial secretion | male | 55 | 0 |  | 1 | 1 |
| CE_297 | Bronchial secretion | male | 69 | 0 |  | 1 | 1 |
| CE_298 | Bronchial lavage | male | 37 | 0 |  | 1 | 1 |
| CE_299 | Bronchial lavage | female | 63 | 0 |  | 1 | 1 |
| CE_300 | Bronchial secretion | male | 76 | 0 |  | 1 | 1 |
| CE_301 | Tracheal secretion | female | 70 | 1 | Underclustering (<100,000 clusters) |  |  |
| CE_302 | Tracheal secretion | male | 69 | 0 |  | 1 | 1 |
| CE_303 | Bronchial secretion | female | 87 | 0 |  | 1 | 0 |
| CE_304 | Bronchial secretion | male | 53 | 0 |  | 1 | 1 |
| CE_305 | Bronchial secretion | female | 74 | 0 |  | 1 | 1 |
| CE_306 | Bronchial secretion | male | 60 | 0 |  | 1 | 0 |
| CE_307 | Bronchial secretion | female | 85 | 0 |  | 1 | 1 |
| CE_308 | Bronchial lavage | female | 63 | 1 | Incomplete evaluation |  |  |
| CE_309 | Bronchial secretion | female | 80 | 1 | Ambiguous species-level identification |  |  |
| CE_310 | Bronchial lavage | male | 79 | 0 |  | 1 | 1 |
| CE_311 | Bronchial lavage | male | 71 | 0 |  | 1 | 1 |
| CE_312 | Bronchial lavage | male | 59 | 0 |  | 1 | 1 |
| CE_313 | Bronchial lavage | female | 63 | 0 |  | 1 | 1 |
| CE_314 | Bronchial lavage | female | 24 | 0 |  | 1 | 1 |
| CE_315 | Tracheal secretion | female | 47 | 0 |  | 1 | 1 |
| CE_316 | Tracheal secretion | female | 78 | 0 |  | 1 | 0 |
| CE_317 | Bronchial secretion | male | 47 | 0 |  | 1 | 1 |
| CE_318 | Bronchial secretion | female | 72 | 0 |  | 1 | 1 |
| CE_319 | Bronchial secretion | male | 58 | 0 |  | 0 | 1 |
| CE_320 | Bronchial secretion | male | 81 | 0 |  | 0 | 1 |
| CE_321 | Bronchial secretion | male | 64 | 0 |  | 1 | 0 |
| CE_322 | Bronchial secretion | male | 65 | 0 |  | 1 | 0 |
| CE_323 | Bronchial secretion | male | 78 | 0 |  | 1 | 1 |
| CE_324 | Tracheal secretion | female | 81 | 0 |  | 1 | 1 |
| CE_325 | Tracheal secretion | male | 1 | 0 |  | 1 | 1 |
| CE_326 | Bronchial secretion | male | 52 | 0 |  | 1 | 0 |
| CE_327 | Bronchial secretion | male | 52 | 0 |  | 1 | 1 |
| CE_328 | Bronchial secretion | male | 72 | 1 | Underclustering (<100,000 clusters) |  |  |
| CE_329 | Tracheal secretion | male | 1 | 0 |  | 1 | 1 |
| CE_330 | Tracheal secretion | male | 70 | 0 |  | 1 | 1 |
| CE_331 | Tracheal secretion | female | 48 | 0 |  | 1 | 1 |
| CE_332 | Bronchial lavage | female | 75 | 0 |  | 1 | 1 |
| CE_333 | Tracheal secretion | male | 73 | 0 |  | 1 | 1 |
| CE_334 | Tracheal secretion | male | 73 | 0 |  | 1 | 1 |
| CE_335 | Tracheal secretion | female | 54 | 0 |  | 1 | 1 |
| CE_336 | Bronchial lavage | male | 74 | 1 | Incomplete evaluation |  |  |
| CE_337 | Bronchial lavage | male | 58 | 0 |  | 1 | 1 |
| CE_338 | Tracheal secretion | male | 65 | 0 |  | 1 | 0 |
| CE_339 | Tracheal secretion | male | 68 | 0 |  | 1 | 1 |
| CE_340 | Tracheal secretion | female | 83 | 0 |  | 1 | 1 |
| CE_341 | Tracheal secretion | female | 24 | 0 |  | 1 | 1 |
| CE_342 | Tracheal secretion | male | 72 | 0 |  | 1 | 1 |
| CE_343 | Tracheal secretion | male | 75 | 0 |  | 1 | 1 |
| CE_344 | Tracheal secretion | female | 82 | 0 |  | 1 | 1 |
| CE_345 | Tracheal secretion | male | 73 | 0 |  | 1 | 1 |
| CE_346 | Tracheal secretion | female | 6 | 0 |  | 1 | 1 |
| CE_347 | Tracheal secretion | male | 0 | 0 |  | 1 | 1 |
| CE_348 | Bronchial lavage | female | 88 | 0 |  | 1 | 1 |
| CE_349 | Bronchial lavage | male | 46 | 1 | Underclustering (<100,000 clusters) |  |  |
| CE_350 | Tracheal secretion | female | 68 | 1 | Underclustering (<100,000 clusters) |  |  |
| CE_351 | Bronchial secretion | female | 48 | 0 |  | 1 | 1 |
| CE_352 | Bronchial lavage | male | 70 | 0 |  | 1 | 1 |
| CE_353 | Tracheal secretion | male | 68 | 0 |  | 1 | 1 |
| CE_354 | Tracheal secretion | male | 69 | 0 |  | 1 | 1 |
| CE_355 | Bronchial lavage | female | 43 | 0 |  | 1 | 1 |
| CE_356 | Bronchial lavage | male | 70 | 0 |  | 1 | 1 |
| CE_357 | Tracheal secretion | female | 54 | 0 |  | 1 | 1 |
| CE_358 | Bronchial lavage | male | 61 | 0 |  | 1 | 1 |
| CE_359 | Tracheal secretion | male | 60 | 0 |  | 0 | 1 |
| CE_360 | Tracheal secretion | female | 54 | 0 |  | 1 | 1 |
| CE_361 | Tracheal secretion | male | 80 | 0 |  | 1 | 1 |
| CE_362 | Tracheal secretion | female | 70 | 0 |  | 1 | 1 |
| CE_363 | Bronchial secretion | male | 73 | 0 |  | 1 | 1 |
| CE_364 | Bronchial secretion | female | 62 | 0 |  | 1 | 1 |
| CE_365 | Bronchial secretion | male | 68 | 0 |  | 1 | 1 |
| CE_366 | Bronchial secretion | female | 75 | 0 |  | 1 | 1 |
| CE_367 | Bronchial secretion | female | 54 | 0 |  | 1 | 1 |
| CE_368 | Bronchial secretion | male | 71 | 0 |  | 0 | 1 |
| CE_369 | Bronchial secretion | male | 64 | 0 |  | 1 | 1 |
| CE_370 | Bronchial lavage | male | 84 | 0 |  | 1 | 1 |
| CE_371 | Bronchial lavage | female | 71 | 0 |  | 1 | 1 |
| CE_372 | Tracheal secretion | male | 77 | 0 |  | 1 | 1 |
| CE_373 | Bronchial secretion | female | 60 | 0 |  | 1 | 1 |
| CE_374 | Bronchial secretion | male | 26 | 0 |  | 1 | 1 |
| CE_375 | Bronchial secretion | male | 26 | 0 |  | 0 | 1 |
| CE_376 | Bronchial secretion | male | 66 | 0 |  | 1 | 1 |
| CE_377 | Sputum | male | 72 | 0 |  | 1 | 0 |
| CE_378 | Tracheal secretion | male | 80 | 0 |  | 1 | 1 |
| CE_379 | Bronchial secretion | male | 60 | 0 |  | 1 | 1 |
| CE_380 | Bronchial secretion | male | 62 | 0 |  | 0 | 1 |
| CE_381 | Bronchial secretion | female | 54 | 0 |  | 0 | 1 |
| CE_382 | Tracheal secretion | female | 70 | 0 |  | 0 | 1 |
| CE_383 | Bronchial secretion | male | 73 | 0 |  | 1 | 1 |
| CE_384 | Tracheal secretion | male | 68 | 0 |  | 1 | 1 |

Supplementary Table 2. Species-level performance using different thresholds of the reported Level of Evidence (LoE) for the positivity of a result. Performance of cultivation is included for comparison purposes.

|  | LoE ≥2 | LoE ≥3 | LoE ≥4 | Cultivation |
| --- | --- | --- | --- | --- |
| Diagnostic Sensitivity | **76.38%** | 65.21% | 35.00% | 53.61% |
| Positive Predictive Value (PPV) | 88.64% | 90.95% | **93.47%** | 100.00% |
| F1 Score | **82.06%** | 75.96% | 50.93% | 69.80% |

Supplementary Table 3. Comparison of clinical Performance parameters of rt-mNGS and cultivation for the positivity of a sample, genus level, species level and species level of clinically relevant organisms. The best value is highlighted in bold type.

| Statistical measure | rt-mNGS | Cultivation |
| --- | --- | --- |
| Positivity of a sample | | |
| Diagnostic Sensitivity | **83.44%** | 78.53% |
| Diagnostic Specificity | **100.00%** | **100.00%** |
| Positive Predictive Value (PPV) | **100.00%** | **100.00%** |
| Negative Predictive Value (NPV) | **86.43%** | 83.09% |
| Negative Likelihood Ratio (LR-) | **0.1656** | 0.2147 |
| Positive Likelihood Ratio (LR+) | **Infinity** | **Infinity** |
| Diagnostic Accuracy | **91.94%** | 89.55% |
| F1 Score | **90.97%** | 87.97% |
| Genus level | | |
| Diagnostic Sensitivity | **75.08%** | 60.18% |
| Positive Predictive Value (PPV) | 96.19% | **100.00%** |
| F1 Score | **84.33%** | 75.14% |
| Species level | | |
| Diagnostic Sensitivity | **76.38%** | 53.61% |
| Positive Predictive Value (PPV) | 88.64% | **100.00%** |
| F1 Score | **82.06%** | 69.80% |
| Species level of clinical relevance | | |
| Diagnostic Sensitivity | **82.86%** | 63.67% |
| Positive Predictive Value (PPV) | 95.31% | **100.00%** |
| F1 Score | **88.65%** | 77.81% |

Supplementary Table 4. Clinical Performance parameters for interim reports (reports 1/6 to 6/6) of the rt-mNGS method.

|  | 1/6 | 2/6 | 3/6 | 4/6 | 5/6 | 6/6 |
| --- | --- | --- | --- | --- | --- | --- |
| Species of clinical relevance | | | | | | |
| Diagnostic Sensitivity | 41.39% | 47.95% | 57.38% | 71.31% | 77.46% | **82.86%** |
| Positive Predictive Value (PPV) | **100.00%** | **100.00%** | 98.59% | 98.86% | 98.44% | 95.31% |
| F1 Score | 58.55% | 64.82% | 72.54% | 82.86% | 86.70% | **88.65%** |
| All Species | | | | | | |
| Diagnostic Sensitivity | 32.09% | 39.79% | 48.66% | 62.89% | 70.70% | **76.38%** |
| Positive Predictive Value (PPV) | 93.75% | **94.66%** | 94.20% | 91.73% | 90.67% | 88.64% |
| F1 Score | 47.81% | 56.02% | 64.17% | 74.62% | 79.45% | **82.06%** |

Supplementary Table 5. Pairwise similarity of the Level of Evidence (LoE) between different types of sequencing devices for 64 evaluated samples. The upper section contains an evaluation for species deemed clinically relevant. The lower section contains an evaluation for all identified species. The agreement represents the percentage of all acceptable differences compared to the total number of identified species.

| Species of clinical relevance | | | |
| --- | --- | --- | --- |
|  | **Acceptable** | | **Unacceptable** |
|  | **LoE difference = 0** | **LoE difference = 1** | **LoE difference ≥ 2** |
| MiSeq™ ↔ MiniSeq™ | 38 (90.48%) | 4 (9.52%) | 0 (0.00%) |
| MiSeq™ ↔ NextSeq™ | 34 (80.95%) | 8 (19.05%) | 0 (0.00%) |
| MiniSeq™ ↔ NextSeq™ | 30 (71.43%) | 12 (28.57%) | 0 (0.00%) |
|  | **Agreement:** 100% | |  |
|  |  | |  |
| All species | | | |
|  | **Acceptable** | | **Unacceptable** |
|  | **LoE difference = 0** | **LoE difference = 1** | **LoE difference ≥ 2** |
| MiSeq™ ↔ MiniSeq™ | 186 (64.36 %) | 96 (33.22 %) | 7 (2.42 %) |
| MiSeq™ ↔ NextSeq™ | 189 (65.40 %) | 90 (31.14 %) | 10 (3.46 %) |
| MiniSeq™ ↔ NextSeq™ | 159 (55.02 %) | 120 (41.52 %) | 10 (3.46 %) |
|  | **Agreement:** 96.89% | |  |
